# Guidewire Ablation via Coronary Venous System for Frequent VPBs: A Case Report

**DOI:** 10.1155/cric/1553244

**Published:** 2026-07-13

**Authors:** Ling Jiang, Jichun Liu, Tao Ge, Jun Tang, Youquan Wei

**Affiliations:** ^1^ Department of Cardiology, The First Affiliated Hospital of Wannan Medical University (Yijishan Hospital of Wannan Medical University), Wuhu, Anhui, China

**Keywords:** drug-refractory arrhythmias, left ventricular summit, percutaneous coronary venous guidewire, radiofrequency ablation, ventricular premature beats

## Abstract

**Background:**

Ventricular arrhythmias originating from the left ventricular summit (LVS) are challenging to treat with conventional catheter ablation due to the region′s complex anatomy, proximity to coronary arteries, and epicardial fat coverage. Percutaneous guidewire ablation via the coronary venous system (CVS) provides a minimally invasive therapeutic option for refractory arrhythmias in this area.

**Case Presentation:**

Herein, we present the case of a 60‐year‐old Asian female patient who had a 1‐year history of palpitations and was diagnosed with symptomatic, frequent ventricular premature beats (VPBs). The patient exhibited an inadequate response to metoprolol and amiodarone as pharmacological therapy and conventional ablation approaches. Following guidewire ablation via the CVS, the VPBs were eliminated entirely, her palpitations fully resolved, and no procedure‐related complications, such as coronary artery injury or thrombosis, were observed. During follow‐up, there was no recurrence of VPBs, and left ventricular function remained normal.

**Conclusion:**

By employing precise mapping, individualized parameter settings, and strict safety monitoring, percutaneous guidewire ablation via the CVS can effectively treat drug‐refractory VPBs originating from the LVS. Importantly, this approach could serve as a complementary option when traditional ablation methods are unsuccessful, offering a feasible therapeutic strategy for complex ventricular arrhythmias.

## 1. Background

The left ventricular summit (LVS) is the most common origin of idiopathic epicardial left ventricular arrhythmias (VAs). The LVS is divided by the great cardiac vein (GCV) into two regions of unequal size. The superior region, owing to its proximity to the coronary arteries and the presence of a thick epicardial fat layer, is generally unsuitable for radiofrequency (RF) ablation, whereas the inferior region is considered relatively safe. The morphological arrangement of the coronary veins in this inferior region also shows considerable variability. Due to its complex anatomy and the limitations of current ablation strategies, catheter ablation in this area is extremely challenging [1]. Ablation within the coronary venous system (CVS) often fails because of the narrow space, proximity to coronary arteries, unstable catheter apposition, and high impedance [2]. To address these challenges, various techniques have been developed, including prolonged endocardial ablation, increased ablation power, bipolar ablation, cryoablation, and surgical ablation under direct vision. Nonetheless, these approaches are often technically complex and carry potential risks, such as myocardial injury [3–7]. For lesions originating from small branches, such as the mid‐to‐distal anterior interventricular vein (AIV), ethanol ablation via the CVS offers distinct advantages. Nevertheless, it requires target vessels of suitable diameter and carries the risk of alcohol leakage [8, 9]. In cases where no clearly suitable target vessel exists or the lesion is located in smaller branch vessels, guidewire ablation can serve as an effective adjunct to ethanol ablation. Herein, we describe a case of drug‐refractory ventricular premature beats (VPBs) originating from the LVS that was successfully treated using percutaneous guidewire ablation via the CVS. This report does not focus on describing a new technology but rather on the individualized procedural experience and key perioperative management points of this case and puts forward a safer, more precise, and minimally invasive treatment option for such conditions, thereby providing practical references for clinically similar challenging cases.

## 2. Case Presentation

A 60‐year‐old Asian female patient was admitted with a 1‐year history of recurrent palpitations. She had a history of well‐controlled hypertension. Transthoracic echocardiography revealed left atrial (LA) enlargement (43 mm), mild dilation of the ascending aorta, mild‐to‐moderate tricuspid regurgitation, and mild aortic regurgitation, with preserved left ventricular systolic function. A 24‐h ambulatory electrocardiogram recorded 23,000 symptomatic VPBs, corresponding to a VPB burden of 23%, which responded poorly to standard treatment with beta‐blockers and other antiarrhythmic drugs. Surface electrocardiography suggested that the VPBs might originate from the left coronary cusp of the aortic valve in the left ventricular outflow tract or from the GCV/LVS region (Figure 1A).

**Figure 1:**
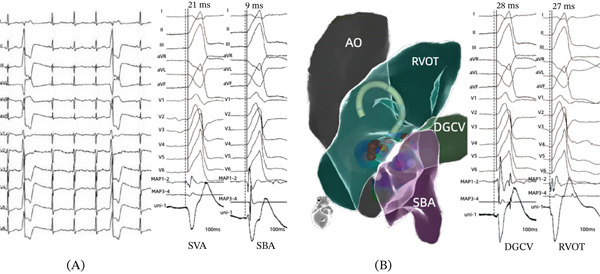
(A) Standard 12‐lead electrocardiogram (25 mm/s) depicting the morphology of premature ventricular contractions (PVCs). (B) Three‐dimensional electroanatomical mapping of the earliest activation sites at supravalvular aorta (SVA), subvalvular aorta (SBA), distal great cardiac vein (DGCV), and right ventricular outflow tract (RVOT, pulmonary valve region).

### 2.1. Electrophysiological Examination and Mapping

After obtaining informed consent, the patient underwent electrophysiological examination. Percutaneous punctures of the right femoral vein and femoral artery were performed. Under the guidance of the three‐dimensional electroanatomical mapping system (CARTO 3), a 3.5 mm tip SmartTouch irrigated ablation catheter was used to sequentially map and ablate the supravalvular aorta (SVA), subvalvular aorta (SBA), distal great cardiac vein (DGCV), and right ventricular outflow tract (RVOT, pulmonary valve region) (Figure 1B) in power‐controlled mode (30–40 W); however, all attempts were unsuccessful. Among these sites, the DGCV showed the earliest activation, preceding the surface electrocardiogram by 28 ms. Mapping of the CVS using a Runthrough guidewire revealed that the earliest ventricular activation site was located at the DGCV, with the local unipolar electrogram preceding the surface QRS complex by 31 ms. Coronary venography demonstrated the absence of a suitable target vessel, precluding ethanol ablation. After a thorough discussion with the patient, it was decided to proceed with percutaneous coronary intervention (PCI) guidewire ablation.

### 2.2. Catheter Manipulation and Ablation

Before guidewire ablation, venography was performed via the coronary sinus to delineate the anatomical structure of the GCV and its branches (Figure 2A,B). A Runthrough guidewire (Terumo Corporation) was then successfully advanced to the distal end of the target GCV with the support of a predilation balloon (1.5 × 10 mm) (Figure 2C). A predilation balloon (Terumo Corporation) was advanced over the guidewire, and the guidewire tip was adjusted to expose only 5 mm for precise mapping. The distal end of the guidewire was connected to the V6 lead of the electrocardiograph via an alligator clip to record unipolar signals (Figure 2D,E). Mapping revealed local activation preceding the QRS complex by 31 ms at the DGCV, along with the presence of a flip potential (Figure 2F).

**Figure 2:**
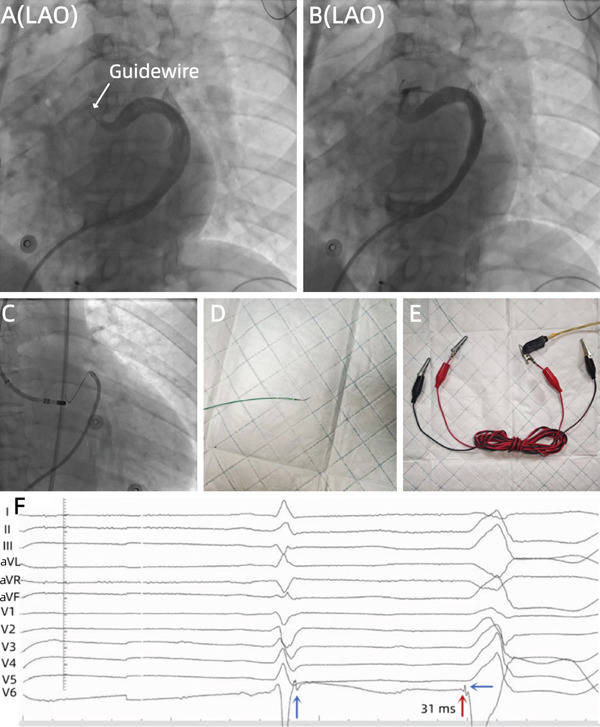
(A, B) Coronary sinus (CS) venogram showing the position of the guidewire. (C) Angiographic image showing the Runthrough guidewire advanced into the left ventricular summit region. (D) Schematic diagram of the guidewire mapping setup. A 0.014‐inch guidewire passing through the balloon. (E) The distal end of the guidewire connected to the V6 lead of the electrocardiograph via an alligator clip. (F) Unipolar recording using a 0.014‐inch Runthrough guidewire showing the local earliest ventricular activation site, preceding the surface QRS complex by 31 ms (red arrow). A characteristic “flip potential” is also visible (blue arrow), indicating a key electrophysiological marker for the origin of VAs.

To minimize the risk of coronary artery injury, intraoperative coronary angiography was performed. Left anterior oblique (LAO) and right anterior oblique (RAO) projections confirmed that the distance between the guidewire tip and major vessels, including the left anterior descending (LAD) and left circumflex (LCX) arteries, exceeded 5 mm (Figure 3A,B). The ablation circuit was established as follows: The guidewire tip was advanced through a microcatheter into the CVS, with the exposed length of the uninsulated portion of the guidewire adjusted according to ablation requirements. The distal end of the guidewire was connected to the ablation catheter, and both were immersed in normal saline to deliver RF energy (Figure 3C,D). Ablation was performed in power‐controlled mode with initial parameters of a 5 mm guidewire exposure length, 10 W output power, and a 3‐s duration. VPBs disappeared within 3 s of ablation initiation (Figure 4); however, a sudden increase in impedance was subsequently observed (dropping from 430 to 350 *Ω* before rapidly rising to 480 *Ω*, followed by current cutoff), suggesting possible microthrombus formation or charring at the guidewire tip. The parameters were then adjusted to a 10 mm guidewire exposure length and an output power of 20 W for reablation. Ablation impedance ranged from 300 to 330 *Ω*, with a cumulative ablation time of 20 s, after which VPBs were eliminated entirely, and the procedure was deemed successful. No further impedance abnormalities occurred, and postprocedural inspection confirmed that the guidewire tip remained intact with no evidence of charring. Isoprenaline was administered to induce VPBs, and no VPBs were observed during a 30‐min monitoring period. A 12‐h postoperative electrocardiogram confirmed the disappearance of VPBs (Figure 5). The patient′s palpitations resolved completely, and no procedure‐related complications occurred.

**Figure 3:**
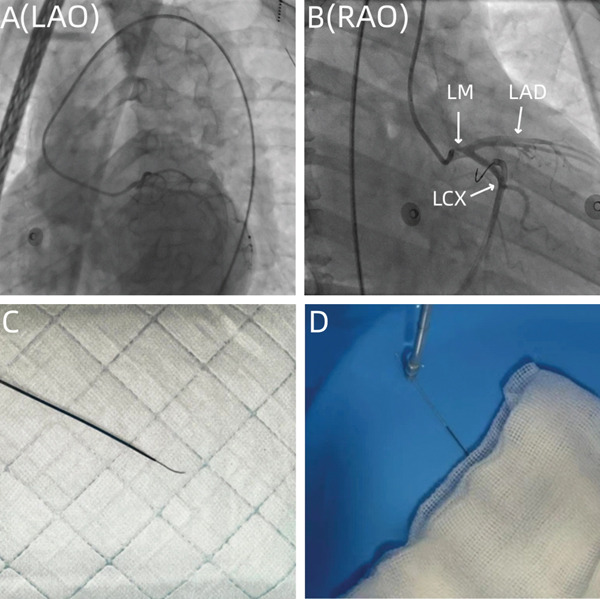
(A) Coronary angiographic images. Left anterior oblique (LAO) view at 35°. LM: left main trunk; LAD: left anterior descending artery; LCX: left circumflex artery. (B) Right anterior oblique (RAO) view at 35°. Both views demonstrate that the distance between the guidewire tip and the main trunk of the left coronary artery exceeds 5 mm. (C) Schematic diagram of the guidewire ablation setup. A 0.014‐inch Runthrough guidewire passing through a microcatheter. (D) The distal end of the guidewire is connected to the ablation catheter, with both immersed in normal saline to deliver radiofrequency energy.

**Figure 4:**
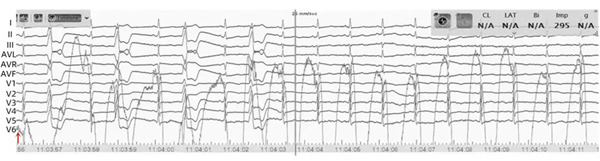
Electrocardiographic recordings during ablation. The red arrow indicates the initiation of radiofrequency delivery. After 3 s of ablation with a 5 mm guidewire exposure and 10 W power, ventricular premature beats (VPBs) disappeared, although an impedance increase was observed. Parameters were then adjusted to a 10 mm exposure and 20 W power, and VPBs were eliminated after a cumulative ablation duration of 20 s.

**Figure 5:**
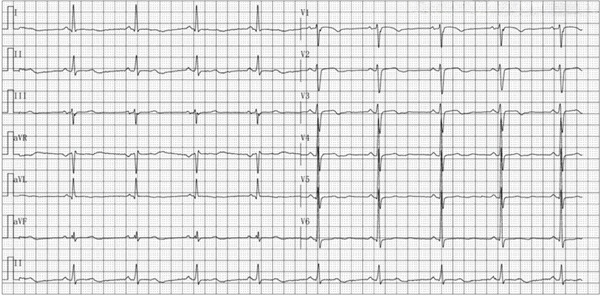
Electrocardiogram (25 mm/s) recorded 12 h after ablation, showing complete disappearance of ventricular premature beats (VPBs).

## 3. Discussion

In the present case, percutaneous guidewire ablation within the CVS was successfully used to treat a patient with drug‐refractory symptomatic VPBs originating from the LVS. Although previous studies have demonstrated the feasibility of guidewire‐based mapping and ablation in the CVS [10, 11], no unified standard has yet been established regarding the optimal guidewire tip exposure length or ablation power [12]. For instance, Efremidis et al. achieved successful ablation using a distally uninsulated guidewire with a 15 mm exposure length at 10–20 W for 20–30 s [13]. Similarly, Wang et al. reported successful outcomes using an uninsulated guidewire with a 25 mm exposed segment covered by a microcatheter, combined with normal saline perfusion at 2 mL/min and delivered at 10–20 W for a total ablation duration of 75 s [14].

The safety of ablation parameters is also crucial. In animal experiments conducted by Wang et al., lesions created with a 10 mm guidewire exposure were larger than those produced with a 20 mm exposure when power and ablation duration were kept constant. Excessively high power settings (e.g., ≥ 20 W) markedly increased the incidence of steam pops, whereas reducing the saline perfusion rate was associated with a lower steam pop incidence. Overall, these findings suggest that using a 10 mm exposed guidewire at 20 W is a relatively safe parameter setting [14]. In our previous in vitro experiments using a porcine heart model, a 10 mm guidewire exposure, 10 W power, and a 10‐s ablation produced an ablation area of 2.4 mm^2^ (impedance of 230 *Ω*) when the ablation catheter was directly connected to the distal guidewire, and both were immersed in normal saline. This was larger than the 1.5 mm^2^ lesion (impedance of 320 *Ω*) generated when the ablation catheter was not connected to the guidewire under the same immersion conditions (Figure S1). Additionally, the incidence of steam pops increased proportionally with increasing power, but the correlation between ablation power and the incidence of steam pops needs to be further verified by large‐sample case series research.

Herein, a Runthrough guidewire was used, with a balloon employed to precisely control the exposed length of the guidewire tip for mapping. For ablation, the guidewire was subsequently covered with a microcatheter, enabling more accurate mapping and lesion delivery. Ablation was performed by connecting the guidewire to the microcatheter and immersing both in normal saline. An initial attempt with a 5 mm exposure and 10 W power resulted in an increase in impedance. After adjusting to a 10 mm exposure length, 20 W power, and a cumulative ablation time of 20 s, complete ablation was achieved without complications, which is broadly consistent with the safety window reported in the literature. We speculate that a power range of 10–20 W combined with a 10 mm guidewire exposure length may represent a relatively safe and effective parameter set for human application.

Another key safety measure in this case was the use of multiprojection coronary angiography during the procedure to confirm that the distance between the guidewire tip and the main coronary artery trunk exceeded 5 mm, thereby minimizing the risk of vascular injury. The guidewire remained intact postoperatively, the electrocardiogram showed no ischemic changes, and the patient reported no discomfort during or after the procedure, further supporting the safety of this technique. However, the applicability of this technique is constrained by anatomical variations of the CVS. Previous studies have shown that only about 12% of idiopathic VAs have their earliest activation within the CVS [15]. Therefore, comprehensive preoperative imaging to assess venous anatomy and precisely localize the target site is essential for procedural success.

Here, we systematically detailed an integrated guidewire‐based mapping and ablation technique within the CVS, offering a replicable procedural strategy for treating refractory VAs originating from the LVS. Notably, during mapping, the exposed length of the guidewire tip was precisely controlled using a balloon. Meanwhile, for ablation, the guidewire was covered with a microcatheter, and the ablation circuit was established through an external connection immersed in normal saline. After multiprojection coronary angiography confirmed a safe separation (> 5 mm) from major coronary arteries, precise mapping and individualized ablation (10–20 W, cumulative 20 s) were successfully performed. All in all, our protocol clearly detailed the device configuration, connection method, and safety precautions, providing substantial clinical reference value.

## 4. Conclusion

In conclusion, guidewire ablation is a safe and effective approach for VAs originating from the CVS, particularly the mid‐to‐distal great cardiac vein/anterior interventricular vein (GCV/AIV), that are challenging to treat with conventional catheters. Nevertheless, intervention success depends on three key factors: establishing precise vascular access, individualizing ablation parameters, and maintaining strict safety monitoring, especially regarding the distance from the coronary arteries. Further prospective studies and long‐term follow‐up are warranted to optimize ablation parameters and evaluate long‐term efficacy.

## Author Contributions

Ling Jiang collected clinical data, conducted a literature review, and drafted the initial manuscript. Jichun Liu participated in the patient′s electrophysiological examination and ablation procedure, assisted with data analysis, and revised the manuscript′s core intellectual content. Tao Ge collected and organized imaging data. Jun Tang supervised the study and finalized the manuscript. Youquan Wei oversaw the clinical implementation of the case, approved the final manuscript, and is responsible for data integrity. Ling Jiang and Jichun Liu contributed equally to this work.

## Funding

This work was supported by grants from the Anhui Provincial Department of Science and Technology (202427b10020019), the Scientific Research Project of Wannan Medical College (WK2024ZQNZ54), and the Project of School‐Enterprise Integration Development Fund (XQZD202415).

## Ethics Statement

Written informed consent was obtained from the patient to use this case and any accompanying images for the purpose of journal manuscript publication. A copy of the written consent is available for review by the journal. Ethical approval was not necessary as this is a case study.

## Consent

Written informed consent was obtained from the patient for publication of this case report and any accompanying images. A copy of the written consent is available for review by the Editor‐in‐Chief of this journal.

## Conflicts of Interest

The authors declare no conflicts of interest.

## Supporting information


**Supporting Information** Additional supporting information can be found online in the Supporting Information section. Figure S1: In vitro experiments on porcine hearts with a power setting of 10 W, a guidewire tip exposure of 10 mm, and an ablation duration of 10 s. (A) The distal end of the microcatheter was closely connected to the tip of the guidewire, forming an integrated assembly, which produced an ablation area of 2.4 mm^2^. (B) The microcatheter and guidewire were placed independently without physical connection, maintaining a distance of approximately 5 mm in the normal saline, resulting in an ablation area of 1.5 mm^2^.

## Data Availability

The data used to support the findings of this study are available from the corresponding authors upon request.
