# Supplementary material for: Guidewire Ablation via Coronary Venous System for Frequent VPBs: A Case Report
Source: Case Rep Cardiol. 2026 Jul 13;2026:1553244. doi: 10.1155/cric/1553244 (PMC13358361; doi:10.1155/cric/1553244)
Supplement: Supplementary file 1 — Supporting Information Additional supporting information can be found online in the Supporting Information section. Figure S1: In vitro experiments on porcine hearts with a power setting of 10 W, a guidewire tip exposure of 10 mm, and an ablation duration of 10 s. (A) The distal end of the microcatheter was closely connected to the tip of the guidewire, forming an integrated assembly, which produced an ablation area of 2.4 mm2. (B) The microcatheter and guidewire were placed independently without physical connection, maintaining a distance of approximately 5 mm in the normal saline, resulting in an ablation area of 1.5 mm2. [file CRIC-2026-1553244-s001.docx]

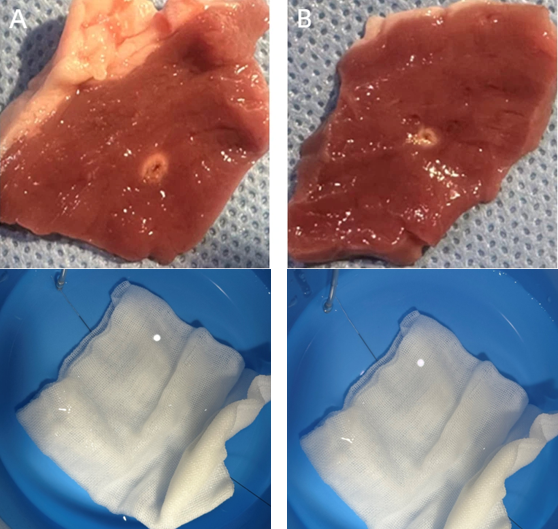


Suppl. Fig. 1. *In vitro* experiments on porcine hearts with a power setting of 10 W, a guidewire tip exposure of 10 mm, and an ablation duration of 10 s. A. The distal end of the microcatheter was closely connected to the tip of the guidewire, forming an integrated assembly, which produced an ablation area of 2.4 mm². B. The microcatheter and guidewire were placed independently without physical connection, maintaining a distance of approximately 5 mm in the normal saline, resulting in an ablation area of 1.5 mm².
